# Application of Microwave Transmission Sensors for Water Cut Metering under Varying Salinity Conditions: Device, Algorithm and Uncertainty Analysis

**DOI:** 10.3390/s22249746

**Published:** 2022-12-12

**Authors:** Kai Zuo, Yi Hong, Haitao Qi, Yi Li, Baolong Li

**Affiliations:** 1 College of Safety and Ocean Engineering, China University of Petroleum-Beijing, Beijing 102249, China; 2 CNOOC EnerTech-Drilling & Production Co., Tianjin 300452, China; 3 CNOOC Research Institute Co., Ltd., Beijing 100010, China; 4 Tsinghua Shenzhen International Graduate School, Tsinghua University, Shenzhen 518055, China

**Keywords:** microwave sensors, microwave transmission line, plane wave theory, iteration algorithm, uncertainty analysis

## Abstract

The measurement of water cut in crude oil is an essential procedure in petroleum production and it is desirable to obtain these data through an automatic and real-time method. Microwave sensors can be used for the task, and they are safe, robust and can cover the whole water cut range. However, they are relatively susceptible to the water conductivity and temperature, and the algorithms for addressing these problems are still rare in the literature. In this paper, a microwave transmission sensor that can measure the water cut under varying salinity conditions is proposed, and the algorithm for solving the water cut and salinity simultaneously with the measured amplitude and phase is described in detail. Experiments under different water cut and salinity conditions are conducted, and the results are used to verify the model and algorithm. Finally, a simplified and fast method for uncertainty analysis is proposed and applied to the iteration algorithm under test conditions. It can be concluded that accuracy higher than 95% in the water cut measurements can be expected under the 0~100% water cut range, and an error of about 10% in the water conductivity is achievable under water-continuous flow conditions. The uncertainty analysis shows that the calculated water cut and salinity results are negatively correlated, and the water salinity uncertainty tends to be larger than the water cut uncertainty. When the water salinity is high, the water cut uncertainty tends to be high whereas the water salinity uncertainty tends to be low.

## 1. Introduction

The measurement of water content in crude oil is an essential procedure in petroleum production and it is usually carried out through a manual sampling and testing method [1]. But this method is known to be time-consuming and inaccurate due to the sampling bias caused by the uneven mixing of oil and water [2]. Therefore, it is desirable to measure the water cut in real time, not only to reduce the time and cost of testing, but also to monitor the operating conditions of each well [3]. Currently, several technologies, such as electrical capacitance/resistance tomography (ECT/ERT) [4,5], gamma-ray [6,7] and ultrasound attenuation methods [8], have been proposed to tackle this problem, but they all bear some limitations to a certain extent. For example, ECT and ERT can only operate normally within the oil-continuous and water-continuous flow conditions, respectively, with the consequence that they cannot cover the whole water cut range [9,10]. The gamma-ray method exploits the density difference between water and oil to determine the water cut [6,7], but its accuracy is relatively limited because the density difference between the oil and water is small. Furthermore, the gamma-ray is harmful to both human bodies and the environment, and its deployment is subject to stringent regulations in most countries. The ultrasound attenuation method uses the attenuation coefficient to determine the water cut [8], but under multiphase working conditions, the propagation of ultrasound will be affected by the gas, which renders this technology unsuitable for multi-phase flow meter (MPFM) applications.

Microwave sensors, however, see a very good contrast between the water and most other materials (e.g., gas and oil), making them well suited for water content measurement in the oil–gas–water multiphase flow. Furthermore, they are functional under the 0~100% water cut range, non-radioactive and insensitive to environmental conditions. As a result, microwave sensors have been employed by many MPFM companies such as Emerson, TechnipFMC and Agar. But microwave sensors suffer from one major problem—they are sensitive to multiple variables (e.g., water salinity and temperature, etc.) besides water cut, which means they should be calibrated separately for a specific application and thus have low universal applicability. Taking water salinity as an example, it directly influences the water conductance and permittivity and, therefore, should be provided as an input in order for the water in liquid ratio (WLR) to be determined uniquely. Because the water salinity is often unavailable in the field, many researchers have proposed different methods to deal with this problem. For example, Emerson employs a separate salinity sensor for this input which operates in the GHz range [11,12]. It is based on a transmission-sensing principle consisting of one transmitting and two receiver antennas. Xie [13,14] proposed a microwave transmission water-cut meter which uses cavity-backed antennas and measures the amplitude attenuation and phase shift at multiple frequencies. The derived mixture permittivity and conductivity are then used to calculate the water cut and salinity, but the details of the iteration algorithm are not given. Sheila-Vadde et al. [15] explored the use of Microstrip patch sensors in transmission mode to estimate the water fraction in saline medium; however, the water salinity should be provided as an input in order for the complex water permittivity to be determined. Zhao et al. [16,17] present a microwave dual frequency correction algorithm that can eliminate the influence of conductivity and obtain the water content. This algorithm uses a deep neural network model to solve the complex nonlinear problem. From the above literature review it can be noted that although the theoretical relationships between the mixture properties and the microwave signals are well known and numerous commercial devices have been proposed to calculate the mixture properties with the measured microwave signals, the detailed iteration algorithm for solving the water cut and salinity simultaneously and the associated uncertainty analysis are still relatively rare in the literature.

This paper first established the theoretical relationships between the mixture properties (e.g., water cut and salinity) and the microwave signals (e.g., phase shift and amplitude attenuation) step by step in Section 2 and then verified the theoretical model with the experimental results. An iteration method for solving the inverse problem was then proposed and the calculated water cut and salinity results were compared with the references to obtain the measurement errors in Section 3. To better analyze the error distributions and improve the measurement accuracy, the uncertainty analysis method was introduced and a novel simplified algorithm was proposed to quickly estimate the uncertainty distributions. The results of the test region are provided in the form of contour maps in Section 4. Finally, important conclusions that can be drawn from the above analysis are summarized in Section 5.

## 2. Theoretical Background

Although the theoretical relationships between mixture properties and microwave signals are generally well known, these equations are usually distributed in different papers and direct data-fitting methods are still widely used in commercial scenarios. Therefore, the theoretical background of the microwave transmission sensors is reviewed here to make the paper more complete, and the connections between the model input X (e.g., water cut and salinity) and the model output Y (e.g., phase shift and amplitude attenuation) are established step by step. These connections are also referred to as the measurement function Y=f(X), and can be used to calculate the propagation of uncertainty in the following sections.

### 2.1. Water Permittivity

Because water is a polar substance, its permittivity $\varepsilon_{wr}$ can be calculated by the Debye relation:

(1) $$\varepsilon_{wr}=\varepsilon_{w\infty }^{\prime }+\frac{\varepsilon_{ws}^{\prime }-\varepsilon_{w\infty }^{\prime }}{1+j\omega \tau }-j\frac{\sigma_{w}}{\omega \varepsilon_{0}}=\varepsilon_{wr}^{\prime }-j\left(\varepsilon_{wr}^{\prime \prime }+\frac{\sigma_{w}}{\omega \varepsilon_{0}}\right)$$

where ω=2πf is the angular frequency, $\varepsilon_{ws}^{\prime }$ is the static permittivity, $\varepsilon_{w\infty }^{\prime }$ is the infinite frequency permittivity, τ is the relaxation time, $\sigma_{w}$ is the conductivity and $\varepsilon_{0}=8.854\times 10^{-12}\;F/M$ is the vacuum permittivity. The $\varepsilon_{wr}^{\prime }$ and $\varepsilon_{wr}^{\prime \prime }$ represent the real and imaginary parts of the permittivity, which can be written as:

(2) $$\varepsilon_{wr}^{\prime }=\varepsilon_{w\infty }^{\prime }+\frac{\varepsilon_{ws}^{\prime }-\varepsilon_{w\infty }^{\prime }}{1+\omega^{2}\tau^{2}}$$

(3) $$\varepsilon_{wr}^{\prime \prime }=\frac{\omega \tau \left(\varepsilon_{ws}^{\prime }-\varepsilon_{w\infty }^{\prime }\right)}{1+\omega^{2}\tau^{2}}$$

Although $\varepsilon_{w\infty }^{\prime }\approx 4.9$ is a constant, the other Debye parameters $\varepsilon_{ws}^{\prime }$, τ and $\sigma_{w}$ are functions of water salinity S, temperature T and microwave frequency f. These functions are based on experimental data and correlations, and can be found in the literature [18,19]. For example, the expressions of $\varepsilon_{ws}^{\prime }$, τ and $\sigma_{w}$ used in this paper are directly adopted from Reference [18] and can be written as:

(4) $$\varepsilon_{ws}\left(N,T\right)=\varepsilon_{ws}\left(T,\;0\right)a\left(N\right)$$

(5) τ(N,T)=τ(T,0)b(N,T)

(6) $$\sigma_{w}\left(T,N\right)=\sigma_{w}\left(25,\;N\right)c\left(N,T\right)$$

where $\varepsilon_{ws}\left(T,\;0\right)$ and τ(T,0) are unary functions of T whereas $\sigma_{w}\left(25,\;N\right)$ is a unary function of N. N denotes the normality of the solution and is a polynomial function of water salinity S, N=f(S).

### 2.2. Brüggeman Mixing Formula

The permittivity of the water–oil mixture is determined by the permittivity, volume fraction and the mixing structure of the water and oil. Several formulas exist to estimate the water–oil mixture permittivity while the Brüggeman formula is a classical and accurate one. For a water-continuous water–oil mixture, the Brüggeman formula can be written as:

(7) $$\alpha_{w}=\frac{\varepsilon_{mr}-\varepsilon_{or}}{\varepsilon_{wr}-\varepsilon_{or}}{\left(\frac{\varepsilon_{wr}}{\varepsilon_{mr}}\right)}^{1/3}$$

whereas for an oil-continuous water–oil mixture, it can be expressed as:

(8) $$\alpha_{w}=1-\frac{\varepsilon_{wr}-\varepsilon_{mr}}{\varepsilon_{wr}-\varepsilon_{or}}{\left(\frac{\varepsilon_{or}}{\varepsilon_{mr}}\right)}^{1/3}$$

where $\alpha_{w}$ is the water cut or water liquid ratio (WLR) and $\varepsilon_{or}$ is the permittivity of oil which can be determined from the Clausius–Mossotti equation:

(9) $$\frac{\varepsilon_{or}-1}{\varepsilon_{or}+2}=C\rho_{o}$$

where $\rho_{o}$ is the density of oil and C is a coefficient that is temperature-dependent. Unlike gas whose density varies with pressure, the oil density is a constant; therefore, $\varepsilon_{or}$ is also considered as a constant in this paper, $\varepsilon_{or}=2.2$.

Equations (7) and (8) can be used to calculate the mixture permittivity $\varepsilon_{mr}$ as long as the water cut $\alpha_{w}$ is known. A cubic equation needs to be solved to obtain the $\varepsilon_{mr}$, and Cardano’s formula is used for this task in this paper.

### 2.3. Plane Wave Theory

In free space, a simple solution of the wave equation is that of a plane wave travelling in the direction of the x-axis. Expressed as a complex number, the plane wave is:

(10) $$E=E_{0}\exp\left(-jk_{s}x\right)$$

where $k_{s}$ is the propagation factor defined as:

(11) $$k_{s}=\omega \sqrt{\mu_{m}\varepsilon_{m}}=k_{0}\sqrt{\mu_{mr}\varepsilon_{mr}}$$

where $\mu_{mr}$ is the permeability, $\mu_{r}=1$; $\varepsilon_{mr}$ is the complex permittivity, $\varepsilon_{mr}=\varepsilon_{mr}^{\prime }-j\varepsilon_{mr}^{\prime \prime }$. If $k_{s}=k_{s}^{\prime }-jk_{s}^{\prime \prime }$ is substituted into Equation (11), then after some simplification, the following expressions can be derived.

(12) $$k_{s}^{\prime }=k_{0}\sqrt{\frac{\varepsilon_{mr}^{\prime }}{2}}\sqrt{1+\sqrt{1+{\left(\frac{\varepsilon_{mr}^{\prime \prime }}{\varepsilon_{mr}^{\prime }}\right)}^{2}}}$$

(13) $$k_{s}^{\prime \prime }=k_{0}\sqrt{\frac{\varepsilon_{mr}^{\prime }}{2}}\sqrt{-1+\sqrt{1+{\left(\frac{\varepsilon_{mr}^{\prime \prime }}{\varepsilon_{mr}^{\prime }}\right)}^{2}}}$$

Equations (12) and (13) show the corresponding relation between the real and imaginary parts of the propagation factor $k_{s}$ and those of the mixture permittivity $\varepsilon_{mr}$. The propagation factor $k_{s}$ in the complex form can also be directly calculated from the complex mixture permittivity $\varepsilon_{mr}$ through Equation (11).

### 2.4. Microwave Transmission Sensors

According to plane wave theory, while propagating in the dielectric materials, the microwaves suffer some amplitude attenuation and phase shift:

(14) $$E=E_{0}\exp\left(-jk_{s}d\right)=E_{0}\exp\left(-k_{s}^{\prime \prime }d\right)\exp\left(-jk_{s}^{\prime \prime }d\right)$$

where $k_{s}$ is the propagation factor, $k_{s}=k_{s}^{\prime }-jk_{s}^{\prime \prime }$, d is the thickness of the sample. $k_{s}^{\prime \prime }$ is the loss factor, and the real term describes the exponential damping with the propagated distance, whereas the imaginary term describes the phase shift.

From Equation (14), it can be noted that compared with the microwave signal transmitted through the air $E_{r}=E_{0}\exp\left(-jk_{0}d\right)$, both the amplitude and the phase of the microwave signal transmitted through the water–oil mixture have changed, and the phase shift and amplitude attenuation can be expressed as:

(15) $$\Delta \phi =\left(k_{s}^{\prime }-k_{0}\right)d$$

(16) $$\Delta A=20\log_{10}\left(\frac{E}{E_{r}}\right)=-\left(20\log_{10}e\right)k_{s}^{\prime \prime }d$$

where the amplitude attenuation is measured in decibels (dB), which are defined as the logarithm of the ratio, as shown in Equation (16).

Equations (15) and (16) show the corresponding relation between the phase and amplitude of the microwave signal and the real and imaginary part of the propagation factor $k_{s}$, from which it can be noted that the corresponding relation is a linear one. It is worth mentioning that since the accurate sample thickness d is unavailable, Equations (15) and (16) are not directly used to calculate the phase and amplitude; instead, linear least squares regressions are used to determine the coefficients, which will be introduced in Section 3.

## 3. Device and Algorithm

After the theoretical model is established and the measurement function Y=f(X) is determined, they must be verified and calibrated with the experimental results before being applied to calculate the water cut and salinity. Therefore, in this section, the experimental devices are introduced first and the experimental data are then used to calibrate the theoretical model. Through this calibration process, the uncertainty of the microwave phase and amplitude u(Y) can be determined. After this, an iteration algorithm for solving the water cut and salinity simultaneously is introduced, which can be written as $X=f^{-1}\left(Y\right)$, and the results are compared with the references to obtain the error distribution.

### 3.1. Experimental Platform and Device

#### 3.1.1. Experimental Platform

**Figure 1:**
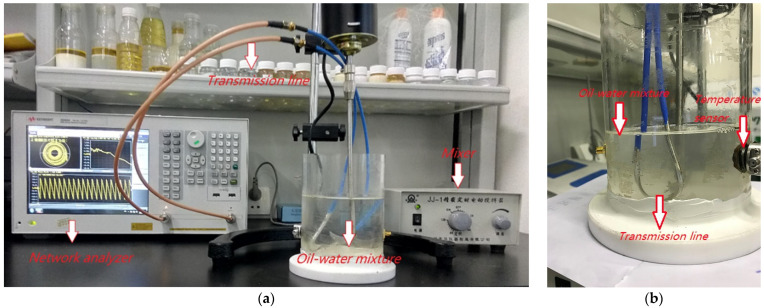
A photograph of the experimental platform and device: (**a**) the experimental platform; (**b**) the microwave transmission device.

A photograph of the experimental platform is shown in Figure 1a. During the experiment, the oil and water are first mixed well in a mixer; the transmission line is then immersed into the oil–water mixture. A microwave network analyzer connected to the transmission line is used to measure the phase shift and amplitude attenuation. A photograph of the microwave transmission device is shown in Figure 1b. The transmission line is modified from a common radio frequency cable which was purchased from Taobao [20]; its product model number is RG 402-141 (SFX-50-3). This RF cable is made of an inner conductor (copper clad steel, coated with silver), a dielectric (PTFE), an outer conductor (copper) and a jacket (FEP). The outer shield layer of a segment is peeled off and the inner conductor is exposed, which is approximately 34 mm in length. The exposed conductor is first bent into a U shape and then completely immersed into the oil–water mixture during the experiment. The RF cable is firmly fixed by a holder and carefully positioned to keep a distance of at least 20 mm off the vessel wall so that the glass wall is outside of the sensitivity region of the transmission line. A thermocouple temperature sensor is inserted into the oil–water mixture from the side of the glass vessel to obtain the exact temperature of liquid. The temperature of the solution is maintained at 40 ℃ throughout this experiment.

At the beginning of the experiment, the transmission line is first fixed and well positioned in the empty glass vessel, and the phase $\varphi_{0}$ and amplitude $A_{0}$ are then recorded by the network analyzer and will be used as the references to calculate the phase shift and amplitude attenuation. During the experiment, the oil–water mixture with specified water conductivity and water cut is first mixed well in the mixer, and then the phase φ and amplitude A of this test point are recorded by the network analyzer. The phase shift and amplitude attenuation are then calculated by $\Delta \varphi =\varphi -\varphi_{0}$ and $\Delta A=A-A_{0}$, respectively.

#### 3.1.2. Microwave Sensor

**Figure 2:**
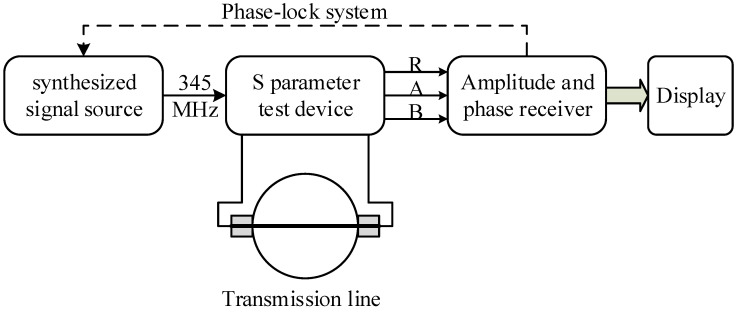
The schematic diagram of the microwave sensor.

**Figure 3:**
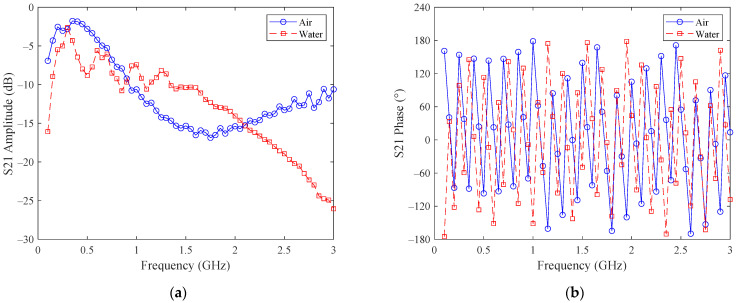
The scattering parameter figure of the microwave transmission system: (**a**) amplitude as a function of frequency; (**b**) phase as a function of frequency.

The schematic diagram of the microwave sensor is shown in Figure 2, from which it can be noted that the microwave sensor consists of a transmission line and a network analyzer, which further consists of a synthesized signal source, a *S* parameter test device, an amplitude and phase receiver, a displayer and a phase-lock system. The microwave signal generated by the synthesized signal source is first divided by the *S* parameter test device, one of which (B) enters the microwave transmission line immersed in the test medium where the amplitude and phase of the microwave signal change accordingly while the other (R) provides references for the amplitude and phase signal. Both signals finally enter the phase and amplitude receiver where the two signals are compared, and the phase shift and amplitude attenuation are obtained. Finally, the *S* parameter is calculated, outputted and displayed. The scattering parameter figure of the microwave transmission system is shown in Figure 3.

Due to the constraint of the hardware conditions, the phase can only be measured at 0~180°, the amplitude can only be measured at 0~40 dB and the frequency is in the range of 300~400 Hz. Based on these factors, iterative simulations are carried out to determine the microwave frequency and sensor size, which are 345 MHz and 34 mm, respectively, in this paper. The permittivity of water, oil and air are roughly 78, 2.2 and 1, respectively; therefore, if the water content of the test medium increases, then its permittivity ε will increase and its wavelength λ will decrease, as shown in the following equation:

(17) $$\lambda =\frac{c}{f\sqrt{\varepsilon }}$$

where ε is the permittivity of the medium, λ is the wavelength, f is the frequency, and c is the speed of light.

Meanwhile, the wavelength of the microwave passing through the shift circuit remains constant, and as a result, the phase shift and amplitude attenuation caused by the wavelength reduction can be used to calculate the water cut and salinity, as explained in Section 2.

### 3.2. Experiments and Data

In this experiment, a total of 55 test points are obtained to cover the water cut range of 0~100% with an incremental step of 10%, and the water conductivity range of 0~4 S/m with an incremental step of 1 S/m. Both the water conductivity $\sigma_{w}$ and the water cut $\alpha_{w}$ can be accurately set, and the phase shift Δϕ and amplitude attenuation ΔA are measured with a microwave network analyzer. With the water conductivity $\sigma_{w}$ and temperature T known, the complex water permittivity $\varepsilon_{wr}$ can be determined by Equation (1). With the water cut $\alpha_{w}$ known, the complex mixture permittivity $\varepsilon_{mr}$ can also be determined by Equations (7) or (8), and the propagation factor $k_{s}$ can be determined by Equation (11). Finally, linear regression fittings are conducted for the Δϕ and ΔA as follows:

(18) $$\Delta \phi =\beta_{p0}+\beta_{p1}k_{s}^{\prime }+e_{p}$$

(19) $$\Delta A=\beta_{a0}+\beta_{a1}k_{s}^{\prime \prime }+e_{a}$$

where $e_{p}$ and $e_{a}$ are the disturbance terms, $\beta_{p0}$ and $\beta_{a0}$ are coefficients. By implementing least square fittings for Δϕ and ΔA, the coefficient $\hat{\beta }$, the covariance matrix $\Sigma_{\hat{\beta }\hat{\beta }}$ and the standard uncertainty of measurand s can be obtained; a more detailed description can be found in Reference [21] and the Appendix A of Reference [22].

**Figure 4:**
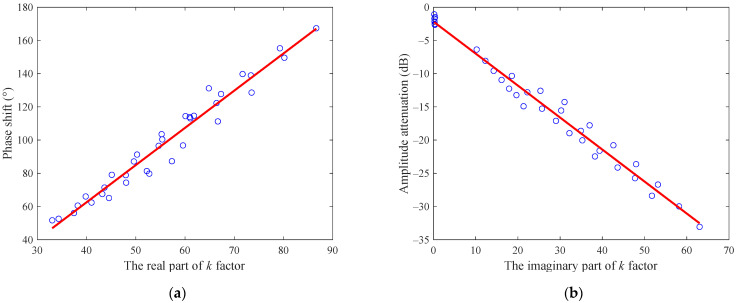
The linear least squares regressions of the microwave phase shift and amplitude attenuation under water-continuous conditions: (**a**) phase shift; (**b**) amplitude attenuation.

The fitting results of the linear least squares regression are shown in Figure 4, from which it can be noted that Δϕ and ΔA are linear functions of $k_{s}^{\prime }$ and $k_{s}^{\prime \prime }$, as predicted by Equations (15) and (16).

### 3.3. Iteration Algorithm for the Water Cut and Salinity Calculation

**Figure 5:**
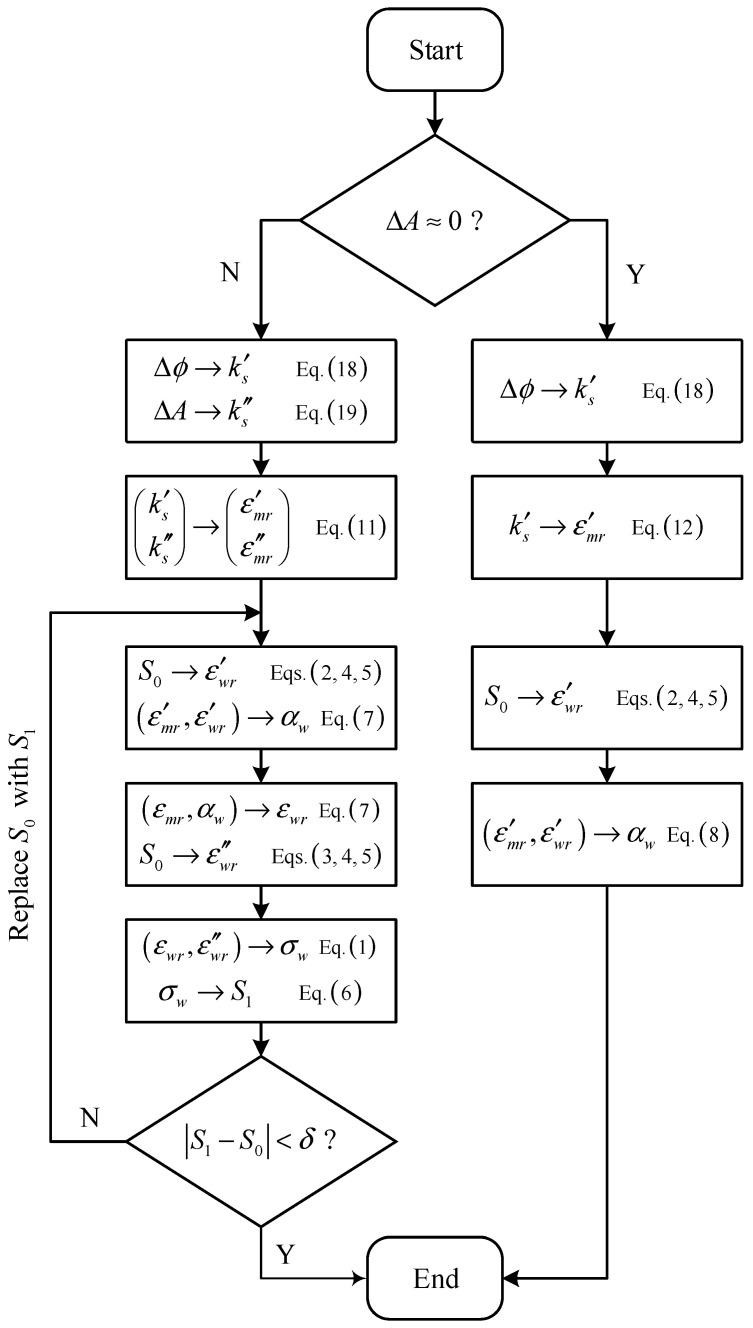
The flow chart of the iteration algorithm for the water cut and salinity calculation.

The flow chart of the iteration algorithm for the water cut and salinity is shown in Figure 5. Because the Brüggeman formula has different forms for the water-continuous and oil-continuous flow conditions, the first step of this algorithm is to use the amplitude attenuation ΔA to determine the flow conditions.

If ΔA is non-zero, then the flow condition is water-continuous, and the procedures of the iteration algorithms are as follows:

1. First, the measured phase shift Δϕ and amplitude attenuation ΔA relative to the air are used to determine the real and imaginary part of the propagation factor $k_{s}$ by Equations (18) and (19) respectively.
2. The propagation factor in its complex form $k_{s}$ is then used to calculate the complex permittivity of the mixture $\varepsilon_{mr}$ by Equation (11).
3. The real part of the mixture permittivity $\varepsilon_{mr}^{\prime }$ is then used to determine the water cut $\alpha_{w}$ by Equation (7). It is worth noting that the water permittivity $\varepsilon_{wr}$ in Equation (7) is a function of water salinity S which is unknown at first. Therefore, an initial value $S_{0}$ is assumed and the real part of the water permittivity $\varepsilon_{wr}^{\prime }$ is calculated from Equations (2), (4) and (5).
4. With the calculated water cut $\alpha_{w}$, the water permittivity in its complex form $\varepsilon_{wr}$ can then be determined by Equation (7) through iterations and the imaginary part of the water permittivity Im(εwr) can thus be obtained.
5. After subtracting the $\varepsilon_{wr}^{\prime \prime }$ calculated by Equation (3), the water conductivity $\sigma_{w}$ can be obtained by Equation (1), and the normality of solution N can be determined by Equation (6).
6. The normality of solution N is then used to update the water salinity, and the updated water salinity $S_{1}$ is substituted into Step 3 to continue the following process until the water salinity converges.

Finally, the water cut $\alpha_{w}$ and water salinity S recorded last time are used as the output.

If the ΔA is near zero, then the flow condition is oil-continuous. For oil-continuous flow conditions, the water cut results are insensitive to the water salinity; therefore, the water salinity S is assumed to be zero and the imaginary part of the water permittivity $\varepsilon_{wr}^{\prime \prime }$ is neglected. Meanwhile, the measured amplitude attenuation ΔA is almost zero, which means that the imaginary part of the propagation factor $k_{s}^{\prime \prime }$ and the mixture permittivity $\varepsilon_{mr}^{\prime \prime }$ are also zero. As a result, the water cut $\alpha_{w}$ can be directly calculated by Equation (8) if the imaginary parts of all parameters are neglected.

### 3.4. Results and Analysis

#### 3.4.1. Water-Continuous Flow Conditions

**Figure 6:**
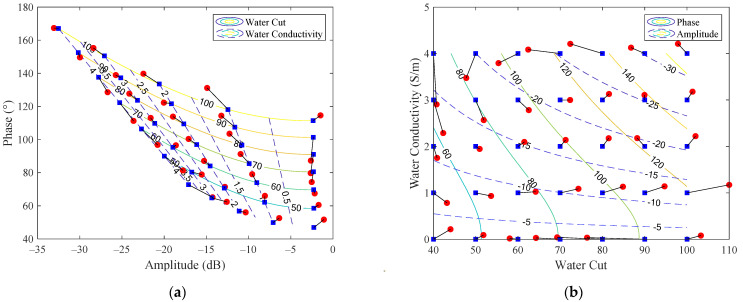
The experimental results (red circles) and theoretical predictions (blue squares) of the 55 test points and the contour maps showing the relationship between (ΔA, Δϕ) and ($\alpha_{w}$, $\sigma_{w}$) predicted by the theoretical model: (**a**) water cut $\alpha_{w}$ and conductivity $\sigma_{w}$ as functions of microwave phase Δϕ and amplitude ΔA, with different contour color lines denoting different $\alpha_{w}$ (solid) and $\sigma_{w}$ (dashed) values; (**b**) microwave phase Δϕ and amplitude ΔA as functions of water cut $\alpha_{w}$ and conductivity $\sigma_{w}$, with different contour color lines denoting different Δϕ (solid) and ΔA (dashed) values. The numbers on each contour color line denote the values of $\alpha_{w}$, $\sigma_{w}$, Δϕ or ΔA, and the length of the black connecting-lines between the red circles and blue squares denote the measurement error values.

The results of the iteration algorithm are shown in Figure 6, where the red circles and blue squares denote the experimental results and theoretical predictions of the 55 test points, respectively, whereas the contour maps show the relationship between (ΔA, Δϕ) and ($\alpha_{w}$, $\sigma_{w}$) predicted by the theoretical model. From Figure 6a, it can be noted that the red measured points (ΔA, Δϕ) deviate from the black theoretically predicted points ($\Delta \hat{A}$, $\Delta \hat{\phi }$) due to the effects of disturbance terms ($e_{a}$, $e_{p}$), as shown by Equations (18) and (19). As a result, the calculated water cuts and conductivities (${\hat{\alpha }}_{w}$, ${\hat{\sigma }}_{w}$) from (ΔA, Δϕ) also deviate from their reference values ($\alpha_{w}$, $\sigma_{w}$), and the differences are referred to as the measurement errors ($e_{\mathrm{wlr}}$, $e_{\mathrm{sig}}$), as shown by the line connecting the red dots and black squares in Figure 6b.

**Figure 7:**
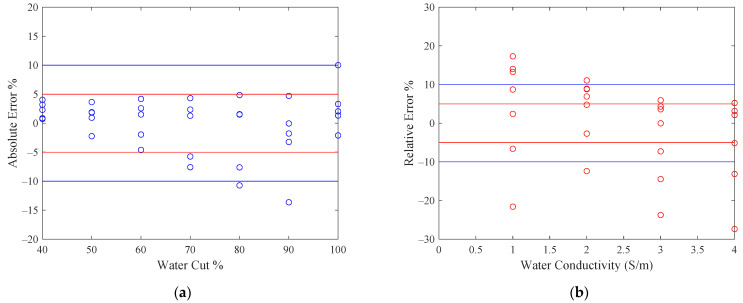
The measurement error of the iteration algorithm and microwave sensor for water-continuous flow conditions: (**a**) the absolute error of water cut; (**b**) the relative error of water conductivity. The red lines denote the ±5% error range and the blue lines denote the ±10% error range.

The measurement errors ($e_{\mathrm{wlr}}$, $e_{\mathrm{sig}}$) of the iteration algorithms are also shown in Figure 7, where the central black line denotes the ideal case with zero error; the upper and lower red lines denote the 5% error range, and the upper and lower blue lines denote the 10% error range. From Figure 7 it can be noted that most of the water cut estimates are within the 5% error range whereas most of the water salinity measurements are within the 10% error range. Therefore, the accuracy of the iteration algorithm and microwave sensor is satisfactory for water-continuous flow conditions.

#### 3.4.2. Oil-Continuous Flow Conditions

**Figure 8:**
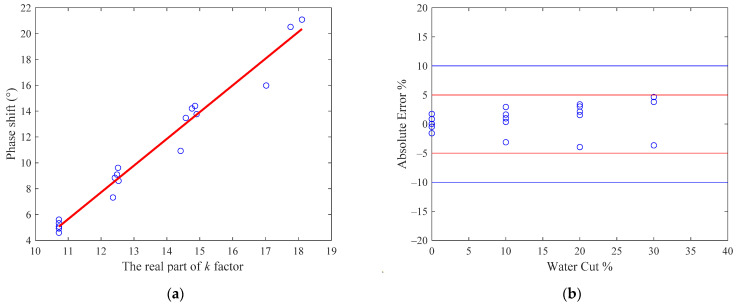
The fitting results of the phase shift and the measurement errors of the water cut under oil-continuous flow conditions: (**a**) the fitting results of the phase shift; (**b**) the measurement error of the water cut. The red lines denote the ±5% error range and the blue lines denote the ±10% error range.

For the oil-continuous flow conditions, the linear least squares regression fitting results of the microwave phase shift is shown in Figure 8a, and the measurement error of the water cut is shown in Figure 8b. From Figure 8b, it can be noted that accuracy higher than 95% in the water cut measurement can also be realized under oil-continuous flow conditions.

## 4. Uncertainty Analysis

In order to further reduce the measurement error and better understand its distributions, it is necessary to conduct uncertainty analysis for this system because the uncertainty and error are related to each other. The uncertainty can be used to predict the error, explain its distributions and track its sources. Therefore, uncertainty analysis is a powerful tool for accuracy improvement, but due to the complexity of the iteration algorithm, the propagation of uncertainty is difficult to calculate analytically and the application of uncertainty analysis is so far limited. In this section, a fast and simplified method for estimating the uncertainty distribution of the water cut and salinity u(X) is proposed, and the results are shown in the form of contour maps. An explanation for this distribution is also provided.

### 4.1. Simplified Method

According to the guidance documents published by the Joint Committee for Guides in Metrology (JCGM), the uncertainty of an output quantity can be evaluated through two methods: one is based on the document “Guide to the expression of uncertainty in measurement” and is referred to as the GUM method for short, and the other is based on its supplements “Propagation of distributions using a Monte Carlo method” and is referred as the Monte Carlo method or MCM method. However, both methods suffer from several problems when it comes to the iteration algorithm: the GUM method requires the model to be linearized and its sensitivity coefficient to be derived, both of which are very difficult for iteration algorithms; whereas the MCM method usually requires huge numbers of calculations, usually in the orders of millions for one test point, which makes it impractical for real-time online applications [22,23]. Therefore, in this paper, a simplified and fast method for evaluating the output uncertainty of the iteration algorithms is proposed.

**Figure 9:**
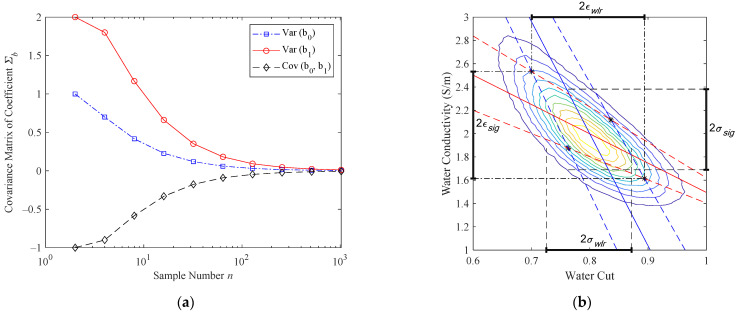
Schematic diagrams of the simplified uncertainty analysis method: (**a**) the coefficient matrix terms as functions of sample number, where the Var(b_0_) denotes the top left term $\frac{1}{n}+\frac{{\bar{x}}^{2}}{S_{xx}}$, the Var(b_1_) denotes the bottom right term $\frac{1}{S_{xx}}$, and the Cov(b_0_, b_1_) denotes the top right or bottom left term $-\frac{\bar{x}}{S_{xx}}$ of Equation (20); (**b**) the output distributions of a test point predicted by the simplified method and the MCM method, where different color lines denote the contour of the joint probability distribution function (PDF) for the MCM outputs, whereas the four asterisks denote the four extreme scenarios of ($\Delta \phi +s_{\Delta \phi }$, $\Delta A+s_{\Delta A}$), ($\Delta \phi +s_{\Delta \phi }$, $\Delta A-s_{\Delta A}$), ($\Delta \phi -s_{\Delta \phi }$, $\Delta A+s_{\Delta A}$) and ($\Delta \phi -s_{\Delta \phi }$, $\Delta A-s_{\Delta A}$) of the simplified method. The ($\sigma_{wlr}$, $\sigma_{sig}$) denotes the standard uncertainty of water cut and salinity obtained by the MCM method, whereas the ($\epsilon_{wlr}$, $\epsilon_{sig}$) denotes the standard uncertainty of the water cut and conductivity obtained by the simplified method.

For unary linear regression, such as Equations (18) and (19), the coefficient matrix $\Sigma_{\hat{\beta }\hat{\beta }}$ can be derived as:

(20) $$\Sigma_{\hat{\beta }\hat{\beta }}=\sigma^{2}\left[\begin{array}{cc} \frac{1}{n}+\frac{{\bar{x}}^{2}}{S_{xx}} & -\frac{\bar{x}}{S_{xx}}\\ -\frac{\bar{x}}{S_{xx}} & \frac{1}{S_{xx}} \end{array}\right]$$

where $S_{xx}=\overset{n}{\underset{i=1}{\sum }}x_{i}^{2}-n{\bar{x}}^{2}$. The variations of each term in Equation (20) with the sample number n are shown in Figure 9a, from which it can be noted that with the sample number n increasing, all three terms of the coefficient matrix $\Sigma_{\hat{\beta }\hat{\beta }}$ decrease to zero. Therefore, in the following simplified analysis, the uncertainties of the coefficients are assumed to be zero due to the large numbers of test points. Meanwhile, the uncertainties of the measurand $s=\sqrt{{\left\|Y-\hat{Y}\right\|}^{2}/\left(n-1\right)}$ will converge to σ; therefore, the standard deviation of the residuals of the ΔA and Δϕ will be used to estimate the output uncertainties.

Figure 9b shows the output distributions of a test point ($\alpha_{w}\;$= 80%, $\sigma_{w}\;$= 2 S/m). The contour map is obtained from the MCM method with the number of runs $M=10^{6}$, while the blue and red lines denote the contour lines of the phase shift Δϕ and amplitude attenuation ΔA, respectively, where the solid lines denote the theoretically predicted values and the dashed lines denote the perturbed values with an additional plus or minus σ. In this test run, the standard uncertainty of the phase and amplitude are set by the s provided by Equations (18) and (19). Therefore, the four asterisks which are the intersections of the four dashed lines delimit the maximum possible area for the output standard uncertainties. As shown in Figure 9b, the standard uncertainties obtained through this simplified method are slightly larger than those obtained with the MCM method, which means it is slightly more conservative than the MCM method, but that it needs many fewer calculations (four in this paper) than the MCM method.

To sum up, in the following simplified uncertainty analysis, the coefficient matrix $\Sigma_{\hat{\beta }\hat{\beta }}$ is assumed to be zero due to the large quantities of test points, and the standard deviation of the residuals of linear regression $s_{\Delta A}$ and $s_{\Delta \phi }$ are used as the perturbations for the measured amplitude and phase, respectively, which are $s_{\Delta A}=1.1835$ and $s_{\Delta \phi }=6.5532,$ respectively.

### 4.2. Uncertainty Distributions

#### 4.2.1. Water-Continuous Flow Conditions

**Figure 10:**
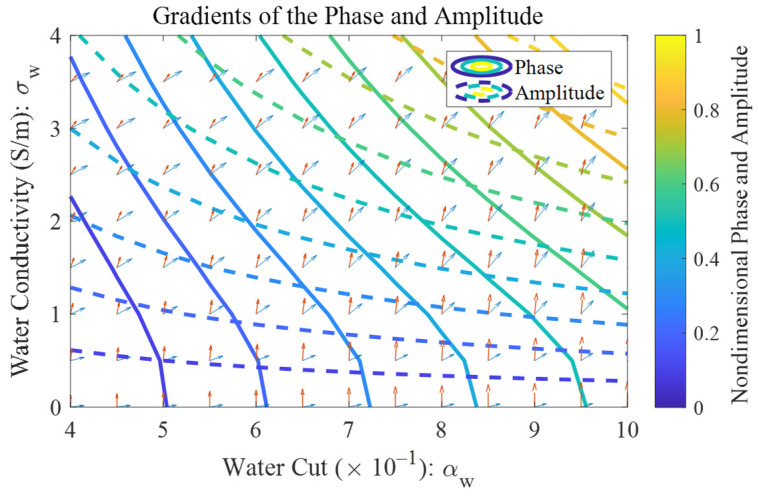
The contour and gradient maps of the phase shift and amplitude attenuation under water-continuous flow conditions. The blue arrows denote the gradient of the phase shift, whereas the red arrow denote the gradient of the amplitude attenuation.

**Figure 11:**
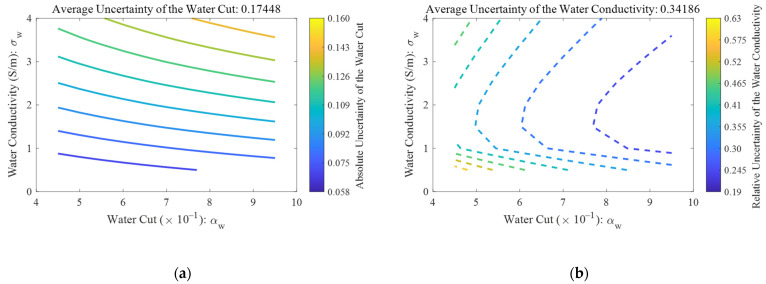
The measurement uncertainty distributions of the microwave network analyzer under water-continuous flow conditions: (**a**) the absolute uncertainty of water cut; (**b**) the relative uncertainty of water conductivity.

From Figure 9b, it can also be noted that as long as the perturbations (or uncertainties) of the phase and amplitude are fixed, the corresponding uncertainties of the water cut and conductivity increase with the intersection angle between the two contour lines (phase and amplitude) decreasing. Therefore, test regions where the contour lines of the phase and amplitude almost overlap will have the largest corresponding uncertainties, which are the top right part of Figure 10, where the contours and the gradients of the phase and amplitude are shown as the solid and dashed lines, respectively. In addition to the intersection angle, the relative uncertainties of the water cut and conductivity are also affected by their mean values, according to the definitions of relative uncertainty $\sigma_{r}=\sigma /\mu$, where μ is the mean value of a test point. Therefore, as the mean value of a test point approaches zero, its relative uncertainty will increase rapidly as a result. The distributions of the relative uncertainties of the water cut and conductivity are shown in Figure 11.

From Figure 11a, it can be noted that the absolute uncertainty of the water cut reaches its maximum in the top right corner where the intersection angles (as shown in Figure 10) are low. The impact of the denominator on the relative uncertainty is shown in Figure 11b, where the water conductivity approaches zero in the bottom of the figure. Therefore, although the intersection angle on the top is always lower than the bottom, the relative uncertainty of the water conductivity reaches its maximum in the bottom left corner.

It can also be noted from Figure 11 that the relative uncertainties of the water conductivity are almost twice as high as those of the water cut, which is in accord with the measurement error distributions in Figure 7. Therefore, with the above-mentioned microwave network analyzer and the iteration algorithm, the output result of the water conductivity tends to be less accurate than that of the water cut.

#### 4.2.2. Oil-Continuous Flow Conditions

**Figure 12:**
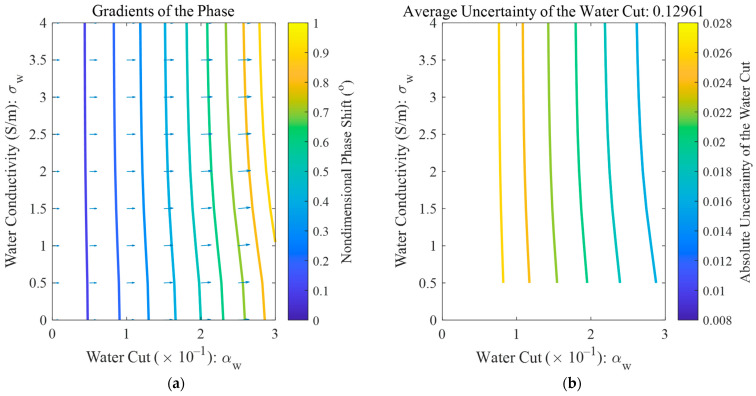
The measurement uncertainty distributions of the network analyzer under oil-continuous flow conditions: (**a**) the absolute uncertainty of water cut, where the blue arrows denote the gradient of the phase shift; (**b**) the relative uncertainty of water conductivity.

For oil-continuous flow conditions, the amplitude of the microwave remains zero, so only the contour and gradient maps of the phase shift are shown in Figure 12a, from which it can be noted that the phase only changes with the water cut and is almost unaffected by the water conductivity. From Figure 12b it can be noted that the absolute uncertainty of the water cut decreases with the water cut increasing, which means that it will be more difficult to measure the water cut accurately under low water cut conditions.

## 5. Conclusions

In this paper, a water cut and salinity metering system based on microwave transmission technology is introduced, and the theoretical relationship between the model input (e.g., water cut and salinity) and the model output (e.g., phase and amplitude) Y=f(X) is established step by step. Experimental data under different water cut and salinity conditions are collected and used to calibrate the theoretical model and obtain the uncertainty of the direct measurements u(Y). Following this, an iteration algorithm for solving the inverse problem $X=f^{-1}\left(Y\right)$ is introduced and the calculated water cut and salinity are compared with the references to obtain its error distribution. Finally, a simplified and fast method for estimating the uncertainty distributions of the water cut and salinity u(X) is proposed, and the results can be used to better analyze the error distribution and track its sources. The following important conclusions could be obtained from this research:

1. With the network analyzer and iteration algorithm, an accuracy higher than 95% in the water cut measurements can be expected under a 0~100% water cut range, and an error of about 10% in the water conductivity is achievable under water-continuous flow conditions.
2. Simplified and fast uncertainty analysis can be carried out for the iteration algorithm, and the results show that the calculated water cut and salinity results are negatively correlated and the output result of the water conductivity $\sigma_{w}$ tends to be less accurate than that of the water cut $\alpha_{w}$.
3. The calculated uncertainty distribution can be used to predict the measurement error, and the results show that the water cut uncertainty reaches its maximum when $\sigma_{w}$ is high, whereas the water conductivity uncertainty reaches its maximum when $\sigma_{w}$ is low.

## Data Availability

Not applicable.
